# Specification of *Drosophila* neuropeptidergic neurons by the splicing component *brr2*

**DOI:** 10.1371/journal.pgen.1007496

**Published:** 2018-08-22

**Authors:** Ignacio Monedero Cobeta, Caroline Bivik Stadler, Jin Li, Peng Yu, Stefan Thor, Jonathan Benito-Sipos

**Affiliations:** 1 Dept. of Biología, Facultad de Ciencias, Universidad Autónoma de Madrid, Madrid, Spain; 2 Dept. of Clinical and Experimental Medicine, Linkoping University, Linkoping, Sweden; 3 Department of Electrical and Computer Engineering Texas A&M University, College Station, Texas, United States of America; 4 TEES-AgriLife Center for Bioinformatics and Genomic Systems Engineering, Texas A&M University, College Station, Texas, United States of America; New York University, UNITED STATES

## Abstract

During embryonic development, a number of genetic cues act to generate neuronal diversity. While intrinsic transcriptional cascades are well-known to control neuronal sub-type cell fate, the target cells can also provide critical input to specific neuronal cell fates. Such signals, denoted retrograde signals, are known to provide critical survival cues for neurons, but have also been found to trigger terminal differentiation of neurons. One salient example of such target-derived instructive signals pertains to the specification of the *Drosophila* FMRFamide neuropeptide neurons, the Tv4 neurons of the ventral nerve cord. Tv4 neurons receive a BMP signal from their target cells, which acts as the final trigger to activate the FMRFa gene. A recent *FMRFa-eGFP* genetic screen identified several genes involved in Tv4 specification, two of which encode components of the U5 subunit of the spliceosome: *brr2* (*l(3)72Ab*) and *Prp8*. In this study, we focus on the role of RNA processing during target-derived signaling. We found that *brr2* and *Prp8* play crucial roles in controlling the expression of the *FMRFa* neuropeptide specifically in six neurons of the VNC (Tv4 neurons). Detailed analysis of *brr2* revealed that this control is executed by two independent mechanisms, both of which are required for the activation of the BMP retrograde signaling pathway in Tv4 neurons: (1) Proper axonal pathfinding to the target tissue in order to receive the BMP ligand. (2) Proper RNA splicing of two genes in the BMP pathway: the *thickveins* (*tkv*) gene, encoding a BMP receptor subunit, and the *Medea* gene, encoding a co-Smad. These results reveal involvement of specific RNA processing in diversifying neuronal identity within the central nervous system.

## Author summary

The nervous system displays daunting cellular diversity, largely generated through complex regulatory input operating on stem cells and their neural lineages during development. Most of the reported mechanisms acting to generate neural diversity pertain to transcriptional regulation. In contrast, little is known regarding the post-transcriptional mechanisms involved. Here, we use a specific group of neurons, Apterous neurons, in the ventral nerve cord of *Drosophila melanogaster* as our model, to analyze the function of two essential components of the spliceosome; Brr2 and Prp8. Apterous neurons require a BMP retrograde signal for terminal differentiation, and we find that *brr2* and *Prp8* play crucial roles during this process. *brr2* is critical for two independent events; axon pathfinding and BMP signaling, both of which are required for the activation of the retrograde signaling pathway necessary for Apterous neurons. These results identify a post-transcriptional mechanism as key for specifying neuronal identity, by ensuring the execution of a retrograde signal.

## Introduction

While transcriptional networks and signalling pathways have been a primary focus during the neural specification processes, relatively little is known about how post-transcriptional modulations control the identity of individual cells. Splicing mechanisms have been widely studied and are known to play an essential role, not only during general mRNA processing, but also as a a regulatory mechanism for generating cellular diversity during development [1–4]. Alternative splicing plays a major part in the generation of protein variation, through mRNA isoform generation, and can also act as a regulatory element, by establishing splicing patterns of batteries of genes specific to certain cell types, tissues or even organisms[5]. Salient examples of alternate splicing patterns stem from *Drosophila*, where sex determination is regulated by dimorphic splicing of pre-mRNAs for *Sex lethal*, *transformer*, *doublesex* and *fruitless* [6]. In addition, splicing factors can specify identity in a tissue, such as the splicing factor *embryonic lethal abnormal vision* (*elav*), which promotes the production of the Ngr^180^ neuroglia-specific isoform in the nervous system, as opposed to the ubiquitous Ngr^167^ isoform [7]. However, to what extent splicing determines specific cell identities is not well understood.

The *Drosophila* Ap cluster is an excellent model for studying mechanisms involved in neural specification in the central nervous system (CNS). The Ap cluster is a well-studied subset of four neurons located laterally in each of the thoracic hemisegments (T1-T3), readily identifiable by the expression of the factors Ap and Eyes absent (Eya). All four Ap neurons are part of the last-born cells of the NB5-6T neuroblast, within which extensive regulatory cascades have been shown to determine the specific fate of each of the neurons of this cluster. These include the Tv1 and Tv4 neuropeptidergic neurons (expressing Nplp1 and FMRFamide, respectively), and also the Tv2 and Tv3 neurons [8–19]. Intriguingly, the Tv4 neuron requires a target-derived retrograde signal, mediated by BMP ligand Glass bottom boat (Gbb) activation of BMP signaling, to terminally differentiate and activate the *FMRFa* gene [11, 20]. This makes FMRFa expression in Tv4 neurons an excellent model for addressing the molecular genetic aspects of retrograde instructive signals during nervous system development.

A recent genetic screen looking for genes involved in Tv4 specification, using an *FMRFa-eGFP* transgene, identified the genes *l(3)72Ab* (FlyBase denomination; herein referred to as *brr2*) and *Prp8* [21]. *brr2* encodes a small ribonucleoprotein (snRNP) type DExD/H Box ATPase with helicase activity, while *Prp8* encodes a PROCT domain protein, both of which associate with the snRNAs U5 complex and are principal components of the spliceosome [22]. Apart from their well-established role in splicing, most notably in yeast, very little is known about Brr2 and Prp8 function. The human ortholog of *brr2*, BRR2 has been described to be involved in retinitis pigmentosa disease (RP) [23, 24], but no role in cell specification.

Here, we address the function of *brr2* and *Prp8*, focusing on *brr2* and its putative role in RNA processing in the acquisition of Tv4 neuronal fate. Our results conclude that *brr2* plays a key and highly specific role controlling the specification of the Tv4 neuron identity. This control is fulfilled by two mechanisms that lead to the activation of the BMP retrograde signaling pathway: (1) correct axonal pathfinding to the target tissue, essential to receive the BMP ligand and, (2) specific RNA processing of the transcripts encoding the BMP receptor subunit Thickveins (Tkv) and Medea, part of the Smad complex, both essential for TGF-β receptor signaling pathway activity. These data demonstrate that specific RNA processing plays a key role in ensuring proper TGFβ/BMP target-derived signalling, and extends our knowledge of the post-transcriptional mechanisms involved in diversifying neuronal identity.

## Results

### Loss-of-function of *brr2* interferes with terminal specification but not the generation of the Ap cluster neurons

Previously, an EMS forward genetic screen using an *FMRFa-eGFP* transgene, a marker for the restricted expression of proFMRFa in the Tv4 neuron, was performed to find key genes involved in the NB5-6T lineage specification. This screen resulted in the identification of several mutants displaying specification defects in the NB5-6T lineage, including two nonsense alleles for the *brr2* gene (FlyBase *l(3)72Ab*), *brr2*^*09C117*^ and *brr2*^*13A036*^, and also one allele of *Prp8* [21]. Here, we focus on the role of *brr2* in the NB5-6T Apterous cluster (Ap cluster) specification at *Drosophila* embryonic stage AFT (Air-Filled Trachea, i.e. 18h after egg laying).

**Fig 1:**
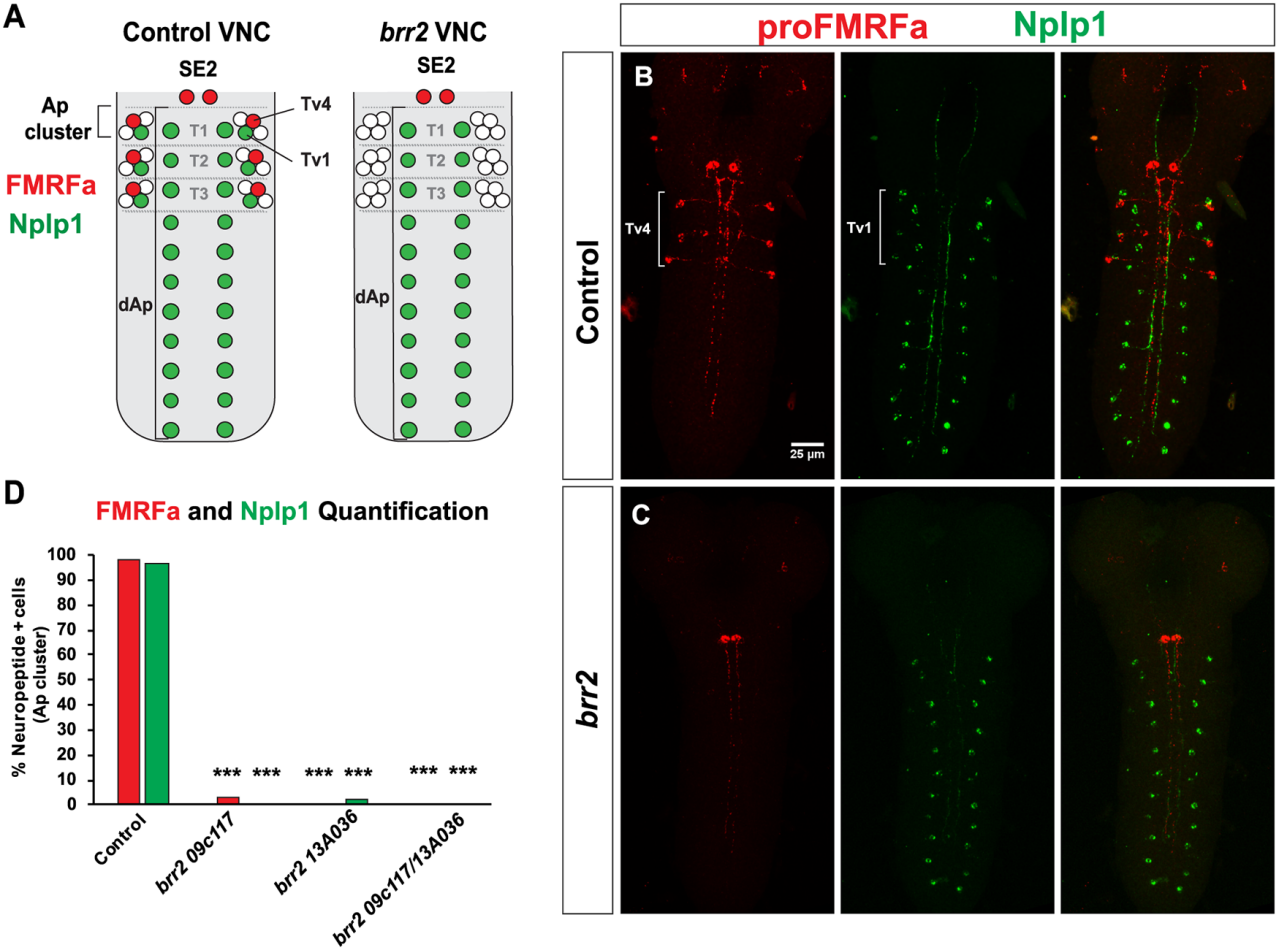
Expression of the Nplp1 and proFMRFa neuropeptides is lost in *brr2* mutants. (A) Schematic representation of proFMRFa and Nplp1 neuropeptide expression pattern in the VNC, in control and *brr2* mutants. (B-C) Immunostaining for proFMRFa and Nplp1 in control (B) and *brr2* mutant (C, *brr2*^*09c117*^*/brr*^*13A036*^) embryo CNS at AFT. (D) Quantification of proFMRFa and Nplp1 expressing cells within the Ap cluster, in control and *brr2* mutants (Pearson’s chi-squared test; n>54 Ap clusters per genotype; *** = p<0.001).

**Fig 2:**
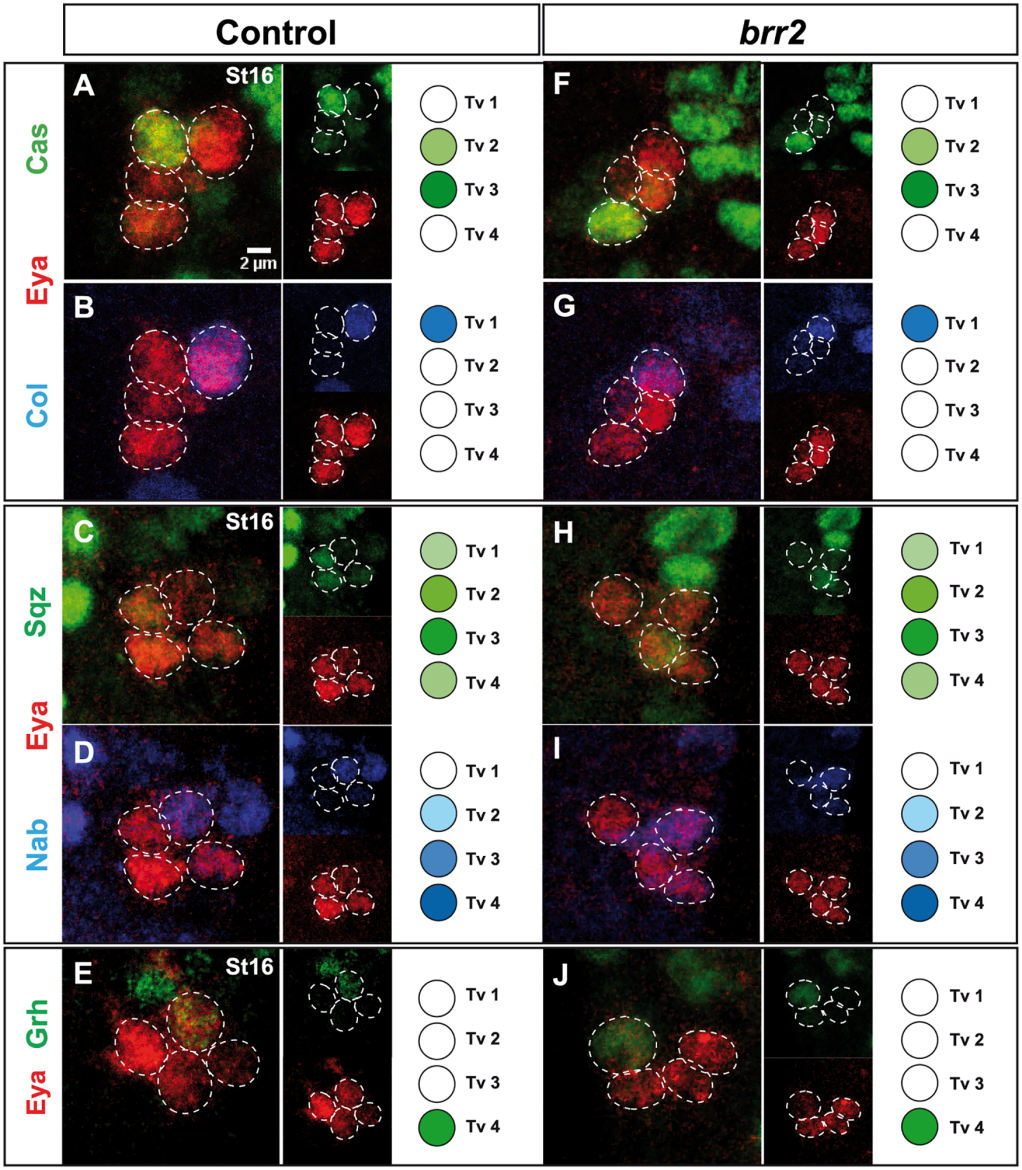
Temporal and sub-temporal regulators of Apterous neuron specification are not affected in *brr2* mutants. (A-J) Expression of Eyes absent (Eya; marking Ap neurons) and the temporal and sub-temporal factors; Castor (Cas), Collier (Col), Squeeze (Sqz), Nab and Grainy head (Grh) in the Ap cluster (2–5 focal plane projections), in control and *brr2* mutants (*brr2*^*09c117*^*/brr*^*13A036*^), at St16. Expression of all five factors appears unaltered in *brr2* mutants, when compared to control.

Initially, we analyzed expression of the terminal markers, proFMRFa and Nplp1 neuropeptides, in *brr2* mutants. These neuropeptides show a highly restricted expression pattern in the VNC. ProFMRFa is expressed in the Tv4 neuron of the Ap cluster (T1-T3) and in two SE2 neurons, while Nplp1 is expressed in the Tv1 neuron of the thoracic Ap cluster (T1-T3) and in the dorsal medial row Ap neurons (dAp neurons, T1-A10; summarized in Fig 1A). Antibody staining of proFMRFa in *brr2* mutants revealed a complete lack of expression of proFMRFa in the Tv4 neurons of the Ap clusters (Fig 1B–1D). Expression of proFMRFa was not affected in the SE2 cells, revealing a restricted function of *brr2* to the Ap cluster. Similarly, antibody staining of Nplp1 in *brr2* mutants revealed the loss of Nplp1 expression in the Tv1 Ap cluster cells, but not in dAp cells (Fig 1C and 1D). The complete absence of both terminal markers and the restriction of the phenotype to the Ap cluster cells in the VNC led us to postulate that Ap cluster cells are not generated, perhaps due to premature cell death or early defects in the NB5-6T lineage. To test this, we analyzed the Ap cluster cells by immunostaining for Eyes absent (Eya), which is present in the four Ap cluster cells at St16 [12]. Eya immunostaining revealed that all Ap cells were present in *brr2* mutants at this stage (Fig 2A–2J).

We conclude that *brr2* mutants generate Ap cluster neurons, but expression of the terminal differentiation markers proFMRFa and Nplp1 is specifically affected. This phenotype is not observed to other proFMRFa- or Nplp1-expressing neurons (SE2 or dAp cells), which suggests a specific role for *brr2* in the determination of neuropeptidergic identity in Ap cluster neurons.

### Temporal and sub-temporal transcriptional cascades of the Ap cluster are not affected in *brr2* mutants

The identity of each cell of the Ap cluster is achieved through an orchestrated expression of transcription factors [8–19]. In order to study the onset of these complex genetic cascades in *brr2* mutants, we performed immunostaining at St16 for key transcription factors, Cas, Col, Grh, Sqz, and Nab. At this stage, Ap cluster cells have not acquired their final identity but the combinatorial expression of these factors allow for the identification of Tv1-Tv4 cells during their specification and early differentiation [8, 13]. We found no apparent alteration in the expression of Cas, Col, Grh, Sqz, or Nab in the Ap cluster cells in *brr2* mutants (Fig 2A–2J). These results revealed that the NB 5-6T lineage progresses normally and correctly generates each of the Ap cluster cells.

### *brr2* is necessary for the expression of Nplp1 in Tv1 and Dimm and pMad in Tv4 neurons.

**Fig 3:**
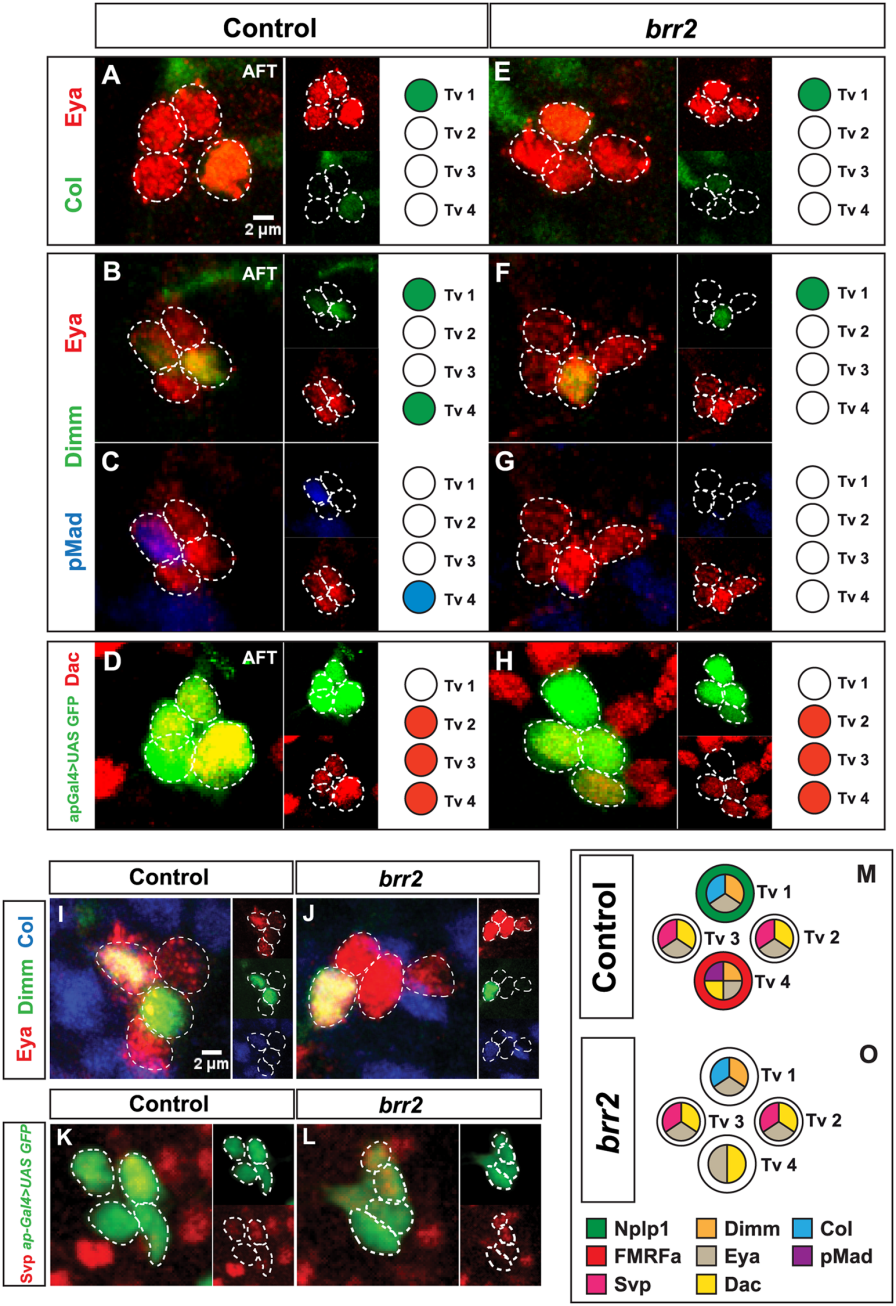
Apterous cell fate determinants are affected in *brr2* mutants. (A-H) Expression of the late factors in the Ap cell fate specification cascade: Eya, Col, Dimmed (Dimm), phosphorylated form of Mothers against dpp (pMad), Dachshund (Dac), and by the reporter line *ap-Gal4>UAS-GFP*, in control and *brr2* mutants (*brr2*^*09c117*^*/brr*^*13A036*^), at stage AFT (2–5 focal plane projections). (I-J) Co-localization of Dimm and Col in Ap clusters (together identifying the Tv1 neuron), in control and *brr2* mutants (*brr2*^*09c11*^*/brr*^*13A036*^) (2–5 focal plane projections). Expression of Dimm is unperturbed in the Tv1 neuron in *brr2* mutants. (K-L) Expression of *ap*, based on the *ap-Gal4>UAS-GFP* reporter GFP expression, and Svp, in the Ap cluster, in control and *brr2* mutant (*brr2*^*09c117*^*/brr*^*13A036*^), at stage AFT. (M-O) Summary of the expression of the late specification factors in the Ap cluster cells (Tv1-Tv4), in control and *brr2* mutants.

Next, to further resolve the *brr2* phenotype, we analyzed the expression of several transcription factors that act during late stages of Ap neuron differentiation, and are necessary for Tv1 and Tv4 neuropeptidergic identities [13]. In the Tv1 neuron, Ap and Eya activate the expression of Dimmed (Dimm), and all three genes, together with Col, trigger Nplp1 expression [13]. In the Tv4 neuron, Ap and Eya also activate Dimm that, together with other two factors, the phosphorylated form of Mothers against dpp (pMad) and Dachshund (Dac), trigger the expression of proFMRFa [11–13]. Therefore, we analyzed the expression of these factors in the Ap cluster cells (Fig 3A–3H).

We did not find differences in expression of either Ap, Eya, Col, and Dac between control and *brr2* mutant embryos (Fig 3A–3H). In contrast, the expression of Dimm was lost in one of the two Dimm-positive cells (Tv1 and Tv4) in all Ap clusters (Fig 3D). Taking advantage of Col immunostaining, which is restricted to Tv1 neurons within the Ap cluster at the AFT stage, we found that Dimm was selectively lost in 100% of Tv4 neurons, in *brr2* mutants (n = 24 Ap clusters) (Fig 3I and 3J).

We previously reported that Svp represses Dimm expression in Tv2 and Tv3 neurons [25], and the timed reduction of Svp expression in Tv1 and Tv4 de-represses Dimm and neuropeptide expression in those neurons by Stg17 [25,26]. Hence, mis-regulation of Svp may explain the absence of Dimm in the Tv4 neuron. However, immunostaining did not reveal differences in Svp expression (Fig 3K and 3L).

In addition to the partial loss of Dimm, pMad expression was almost completely abolished in *brr2* mutants (97% n = 49; p<0,001) (Fig 3F). Segments T1, T2 and T3 were similarly affected. However, pMad staining could be still detected in other cells outside the Ap cluster (S1A–S1F Fig), demonstrating that there was no general defect on the phosphorylation of pMad. To further address if the BMP signaling defect observed in *brr2* mutants was limited to the Ap cluster we analyzed another peptidergic neuronal subtype, the Insulin-like peptide 7 (Ilp7) neurons, which also require BMP signalling for the proper Ilp7 expression [27]. Surprisingly, both Ilp7 and pMad expression was completely unaffected in these neurons in *brr2* mutants (S2B and S2D Fig). Additionally, we quantified the total number of pMad-positive cells in thoracic and abdominal segments in *brr2* mutants and did not find significant differences when compared to control (S1A–S1F Fig).

Our results demonstrate that *brr2* is necessary for the expression of pMad, Dimm and proFMRFa in Tv4 neurons, and for Nplp1 in Tv1 neurons.

### Axonal pathfinding of Tv4 neurons is disrupted in *brr2* mutants

Tv4 neurons project their axons towards the midline and exit the VNC at the dorsal midline, to innervate a peripheral secretory gland; the dorsal neurohemal organ (DNH). When the axon reaches the DNH, it receives the TGFβ/BMP ligand Glass bottom boat (Gbb), which results in the phosphorylation of Mad (pMad), triggering the expression of the *FMRFa* neuropeptide gene[11–20]. Therefore, the absence of pMad in *brr2* mutants could be due to defects at different points of this process, including problems with BMP signaling, as well as axon pathfinding problems.

**Fig 4:**
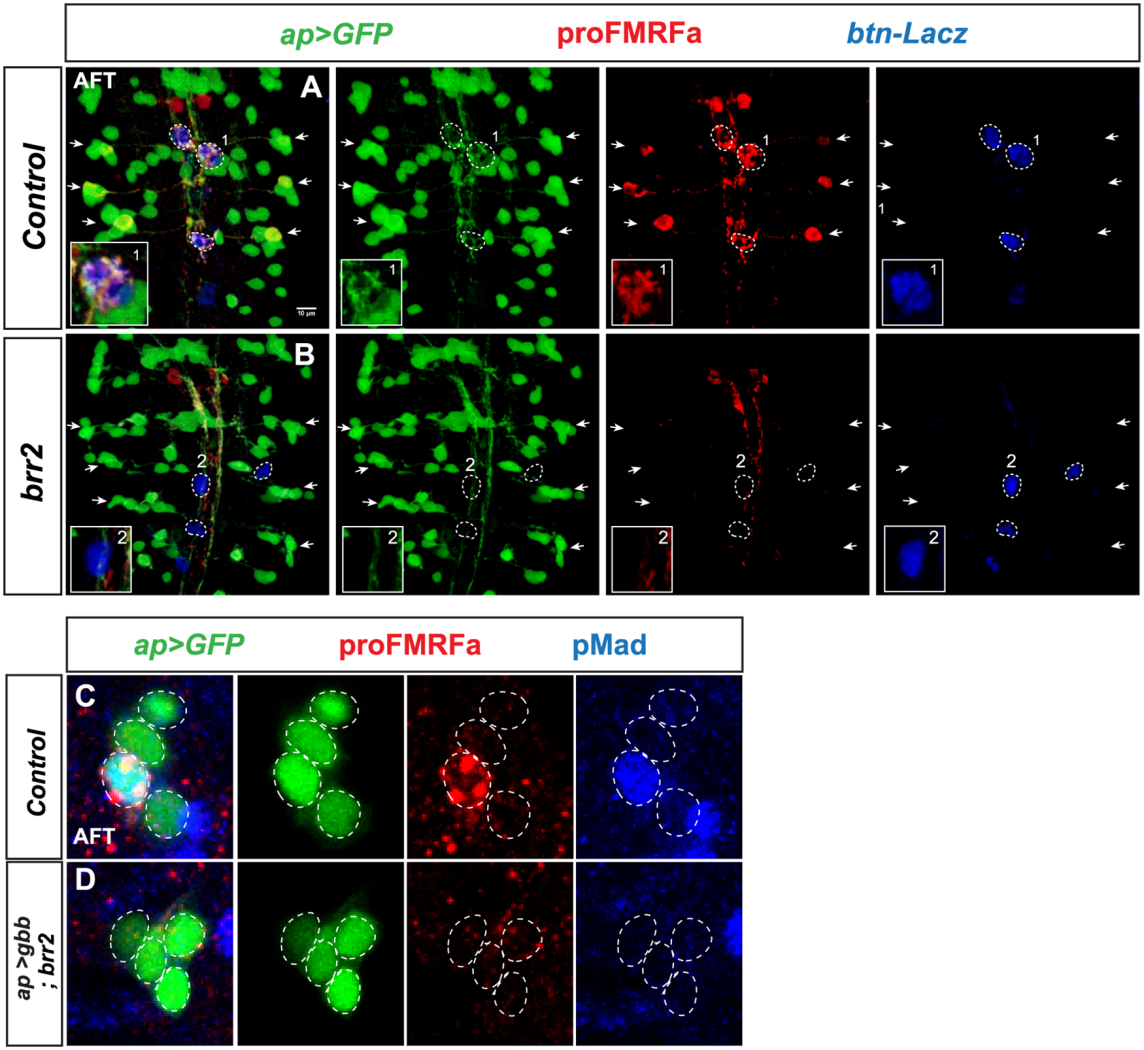
Axonal pathfinding and BMP pathway activation are affected in *brr2* mutants. (A-B) Expression of proFMRFa, *ap-Gal4>UAS-GFP* and *buttonless-lacZ* (*btn>lacZ*), in control and *brr2* mutant (*brr2*^*09c117*^) VNCs, at stage AFT (thoracic segments T1-T3). (A) In control, the six lateral Tv4 neurons project their axons (GFP- and proFMRFa-positive) into the three dorso-medial DNHs (*btn>lacZ* expressing; dashed circles). Box inset shows close-up on one DNH. (B) In *brr2* mutants, the three DNHs are present, but Tv4 neurons fail to innervate the organ. (C-D) Transgenic expression of *gbb* in the Ap cluster of *brr2* mutants *(ap>gbb; brr2*^*09c117*^*)* fails to rescue proFMRFa and pMad (2–5 focal plane projections; stage AFT).

Because proFMRFa is absent in *brr2* mutants, the lack of markers prevented us from identifying the Tv4 neuron and follow its axon trajectory. Thus, in order to address possible defects in the axonal pathfinding of Tv4 neurons during its innervation of the DNH, we monitored the axon pathfinding of the Ap cluster through the expression of *CD8*::*GFP* under the promotor of *ap* gene, using the Gal4-UAS system [28] in combination with the reporter *buttonless-lacZ* (*btn-LacZ)*, which identifies the DNH. 3D analysis of this staining revealed that while Tv4 always reached the DNH in controls it failed to do so in *brr2* mutants (Fig 4A and 4B and S2 Video). These results indicate that in *brr2* mutants, Tv4 axons fail to receive the Gbb ligand and therefore fail to phosphorylate Mad.

### BMP receptor function in Tv4 neurons is disrupted in *brr2* mutants

Previous studies described the failure of Mad phosphorylation upon a failure of axonal pathfinding to the DNH, in *sqz* mutants and upon misexpression of *robo* in Ap-neurons [11]. In both cases, ectopic expression of *gbb* in Tv4 rescued Mad phosphorylation and proFMRFa expression. In order to elucidate if BMP signaling is not triggered in *brr2* mutants merely due to defective axonal pathfinding, we ectopically expressed *gbb* in the Ap cluster of *brr2* mutants using the *ap-Gal4* driver (*ap>gbb)*, and performed immunostaining of pMad and proFMRFa (Fig 4I–4P). Surprisingly, we found that expression of Gbb in the Ap neurons failed to restore pMad or proFMRFa expression in *brr2* mutants. This suggested that expression and/or processing of other components in the BMP signaling pathway may be affected.

**Fig 5:**
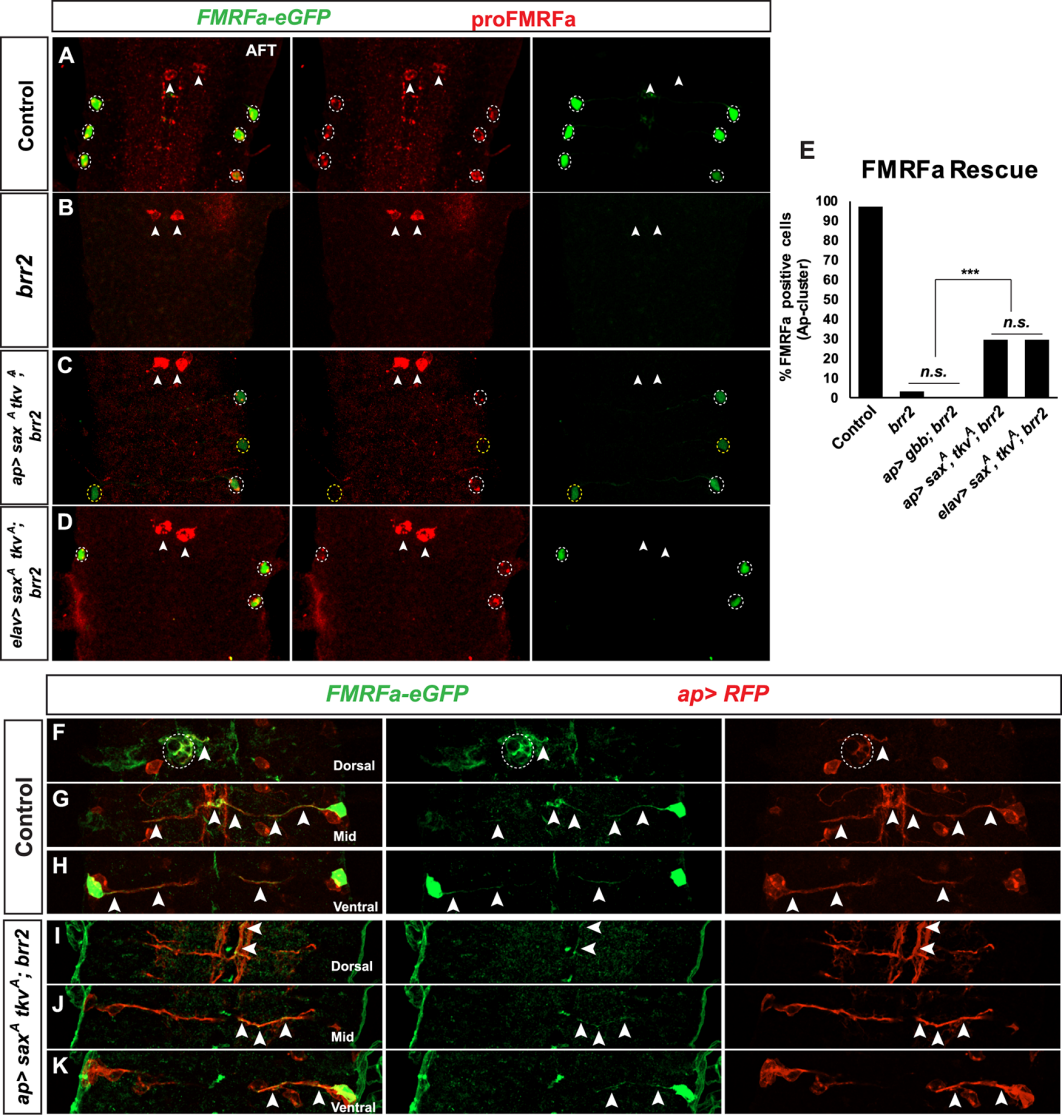
Rescue of proFMRFa expression in *brr2* mutants by ectopic expression of active forms of BMP receptor. (A-D) Expression of *FMRFa-eGFP* and proFMRFa, in control, *brr2* mutants, and *brr2* mutants rescued by transgenic expression of activated BMP receptors (*sax*^*A*^ and *tkv*^*A*^), at stage AFT (thoracic segments T1-T3). (A) In control, the six Tv4 neurons express both *FMRFa-eGFP* and proFMRFa. The SE2 neurons (arrows) express proFMRFa but not *FMRFa-eGFP*, due that this reporter uses a Tv4-specific enhancer [21, 32]. (B) In *brr2* mutants (*brr2*^*09c117*^) expression of proFMRFa and *FMRFa-eGFP* expression is lost in Tv4 neurons, while proFMRFa is not affected in SE2 neurons. (C-D) Transgenic expression of *sax*^*A*^ and *tkv*^*A*^ in *brr2* mutants (*brr2*^*09c117*^), from *ap-Gal4* (C) or *elav-Gal4* (D) results in partial rescue of *FMRFa-eGFP* and proFMRFa (double-positive cells = white dashed circle; GFP-positive cells only = yellow dashed circle). (E) Quantification of *FMRFa-eGFP* expressing cells within the Ap cluster in control, *brr2* mutants, *ap>gbb;brr2*, *ap>sax*^*A*^ *tkv*^*A*^*;brr2* and *elav>sax*^*A*^ *tkv*^*A*^ (Pearson’s chi-squared test, n>47 Ap clusters per genotype; *** = p<0.001; n.s = non-significant). (F-K) Expression of *FMRFa-GFP* and *ap>RFP*, identifying the Tv4 cell body and its axon, in control and *ap>sax*^*A*^ *tkv*^*A*^*; brr2* mutants (*brr2*^*09c117*^), at stage AFT (dorsal, mid and ventral views of T2 segment of the VNC). (F-H) In control, the Tv4 axon (arrows) project to the midline, and then dorsally into the DNH (dashed circle). (I-K) In *ap>sax*^*A*^ *tkv*^*A*^*;brr2*, rescued mutants, Tv4 cells project their axon normally towards the midline, but fails to project dorsally into the DNH. Instead, the axon follows the same trajectory as the rest of the Ap cluster neurons (*ap>RFP*), projecting ipsilaterally and anteriorly.

The BMP signaling pathway is triggered by binding of Gbb to a hetero-tetrameric receptor complex, formed by Wishful thinking (Wit), Saxophone (Sax) and Thickveins (Tkv), and Tkv/Sax-kinase activity to phosphorylate Mad, which is transported to the nucleus to act as a transcription factor, in a complex with the co-Smad, Medea [29]. To address if BMP receptor expression/assembly was affected in *brr2* mutants, we ectopically expressed constitutively active forms of Sax (*UAS-sax*^*A*^) and Tkv (*UAS*-*tkv*^*A*^) [30], previously shown to restore proFMRFa expression in mutants of BMP signaling pathway receptors [11,31]. To this end, we used two different drivers: *elav-Gal4* (*elav>sax*^*A*^, *tkv*^*A*^*; brr2)* and *ap-Gal4* (*ap-Gal4> sax*^*A*^, *tkv*^*A*^*; brr2*). We observed a partial rescue of *brr2* mutants in both cases (29.8% n = 84 p<0.001 and 29.8% n = 47 P>0.001, respectively) (Fig 5A–5D). This suggested that defects in receptor expression/assembly may in part underlie the *brr2* phenotype. Nevertheless, the rescue was less robust than anticipated. This could potentially explained by *FMRFa* mRNA processing defects, partial rescue of Mad phosphorylation or perhaps Dimm expression.

To confirm that *brr2* affected transcription of the *FMRFa* gene, as opposed to the processing and translation of the *FMRFa* mRNA, we analyzed the expression of the *FMRFa-eGFP* reporter gene; a construct with the previously identified FMRFa Tv enhancer of 446 bp inserted upstream of eGFP [21, 32]. We found that expression of eGPF was lost hand-in-hand with the proFMRFa immunostaining (Fig 5B).

We postulated that the lack of Dimm, observed in Tv4 neurons in *brr2* mutants, may underlie the partiality of the rescue. However, further analysis of the rescue did not reveal any correlation between the presence of Dimm and the rescue of proFMRFa (S4D and S4E Fig). Indeed, we observed partial rescue of Dimm expression in *ap-Gal4> sax*^*A*^, *tkv*^*A*^*; brr2* (63% n = 82 clusters p>0,001, S4F Fig). With regards pMad, we found that pMad was rescued in the majority of Tv4 neurons, in *ap-Gal4> sax*^*A*^, *tkv*^*A*^*; brr2* embryos (89% n = 47 P> 0,001, S4I Fig). Thus, a lack of pMad rescue did not fully account for the partial proFMRFa rescue.

The rescue of proFMRFa expression in a subset of Tv4 neurons, in *ap-Gal4>sax*^*A*^, *tkv*^*A*^*; brr2* embryos, allowed us to track those axons in *brr2* mutants. We found that the Tv4 axon initially projects normally towards the midline, but subsequently fails to project dorsally into the DNH. Instead, it follows the same trajectory as the rest of the Ap cluster neurons, projecting ipsilaterally and anteriorly (Fig 5F–5K, S3 and S4 Videos).

Our results reveal that *brr2* has a crucial role in the specification of Tv4 identity through both axonal pathfinding and BMP signaling, both of which are essential for proFMRFa neuropeptide expression.

### BMP receptor activity is interrupted in Tv4 by an impairment of splicing

Brr2 is an important component of the U5 subunit of the spliceosome. To address whether the defects observed in *brr2* mutants were indeed related to a defect in splicing activity, we analyzed *Prp8*, which encodes another component of the U5 subunit of the spliceosome and has been described as a regulator of Brr2 in mRNA processing [22, 33–35]. *Prp8* was also identified in the *FMRFa-eGFP* genetic screen as a gene showing loss of eGFP expression [21]. If the phenotype of *brr2* in Tv4 neurons is due to its splicing activity, we expected to find a phenocopy in *Prp8* mutants. To this end, we performed immunostaining of proFMRFa, Dimm and pMad in *Prp8* mutants at AFT. Our results revealed an absence of proFMRFa specifically in Ap cluster (S6A–S6C Table). The loss of Dimm and pMad expression in *Prp8* mutants resembled that found in *brr2* mutants (S5 Fig).

**Fig 6:**
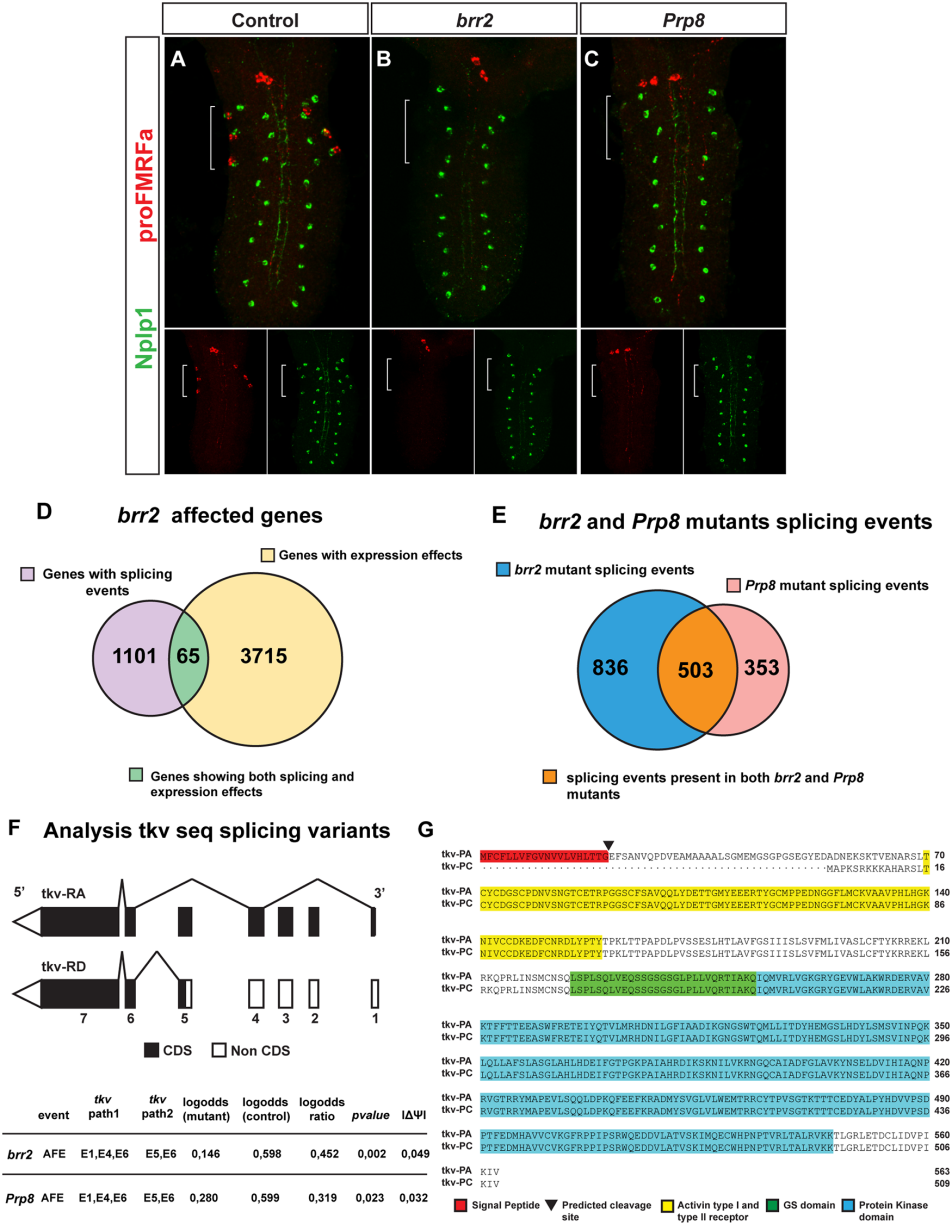
BMP pathway components are affected by impaired splicing. (A-C) Expression of proFMRFa and Nplp1 in control (A), *brr2* mutants (B) and *Prp8* mutants (C). Both *brr2* and *Prp8* mutants display loss of proFMRFa and Nplp1 expression in the Ap cluster, while SE2 and dAp neurons are unaffected. (D) Venn diagram summary of genes affected in *brr2* mutants, as revealed by RNA-Seq transcription and splicing analysis. (E) Venn diagram summary of splicing events detected in both *brr2* and *Prp8* mutants. (F) Schematic representation of splicing events of *tkv*, detected in both *brr2* and *Prp8* mutants. (G) Comparison of the deduced amino acid sequence of the Tkv-PA protein isoform (deduced from the *tkv-RA* mRNA transcript) and tkv-PC (deduced from *tkv-RD)*. The signal peptide (red, SignalP-4.1 software prediction) is present in Tkv-PA but not in Tkv-PC, which is the isoform that is more abundant in *brr2* and *Prp8* mutants. Tkv-PA and Tkv-PC do not differ in other protein domains.

The phenocopy of *brr2* and *Prp8* mutants suggested that Tv4 cell differentiation defects result from their roles as components of the spliceosome. The selective effect of *brr2* and *Prp8* on Ap neurons stands in contrast to their described roles as essential and ubiquitous components of the spliceosome [36, 37] (Figs 1C and 5). This prompted us to attempt to unravel which specific genes were responsible of the defective differentiation of the Ap cluster neurons. To this end we performed RNA-Seq analysis of RNA extracted from control, *brr2* and *Prp8* St15-AFT embryos. The RNA-Seq data were analyzed both for splicing aberrations and for gene expression changes. Focusing first on splicing aberrations, our results (S1–S5 Tables) revealed 1338 significantly altered splicing events (|ΔΨ| > 0.03 and *q* < 0.25) in *brr2* and 855 *Prp8*, with 512 being common to both mutants (Fig 6D and 6E; S1 Table). Among the common genes identified, only the BMP receptor *tkv* is known to play a role in Ap cluster specification. Specifically, we found that *tkv* shows defective splicing involving an alternative first exon (AFE) in both *brr2* and *Prp8* mutants (|ΔΨ| = 0.03163; qvalue = 0.1448; pvalue = 0.0234). In the case of *brr2*, the AFE path E5-E6 is favored, which is predicted to generate the shorter protein isoform tkv-PD (509 aa). The PD isoform generates a protein with unaltered Tkv transmemebrane and kinase domains but lacking the signal peptide (Fig 6F). This protein isoform is less efficient in its ability to bind ligand and may be involved in dosage-dependent tkv-ligand interactions [38]. The RNA-seq results showed that isoform tkv-PA was still present in *brr2* mutants, but since the RNA was extracted from whole embryo it remains unclear whether or not Tv4 neurons express both isoforms.

In addition, in *brr2* mutants, but not in *Prp8*, we also found defective splicing of Medea. Medea encodes an ortholog of the co-smad Smad4, which complexes with phosphorylated Receptor Smads (Smad1,5,9 in vertebrates and Mad in *Drosophila*), to form the Smad complex which translocates to the nucleus to act as transcriptional regulator. In the case of *brr2* mutants, the Exon Skipped (ES) E4 is favored, which generates the Med-RB isoform where the fourth exon is absent. *In vitro* studies have shown that the presence or absence of the alternative fourth exon does not affect Medea binding activity [39]. Nevertheless, the behavior of *Medea* isoforms *in vivo* is unknown. *Medea* amorphic alleles that affect all isoforms eliminate proFMRFa expression in Tv4 neurons but roles for specific isoforms have not been rigorously discriminated (personal communication Anthony Berndt and Douglas Allan).

In order to determine if pMad is properly transported into the nucleus, we analyzed the co-localization of pMad antibody and the nuclear marker DAPI in *ap-Gal4>sax*^*A*^, *tkv*^*A*^*; brr2* genetic background. We found an apparently unaltered nuclear localization of pMad (S4G and S4H Fig). Nevertheless, we cannot rule out that the mis-spliced Medea variant affects DNA-binding efficiency, and hence, defective splicing of Medea could be contributing to the lack of proFMRFa found in *brr2* mutants. Indeed, the limited efficiency of the genetic rescue suggests a contribution of Medea to the *brr2* phenotype.

We performed Gene Ontology (GO) enrichment analysis in order to elucidate if *brr2* mutant splicing defects are more prevalent in neuronal specification due to the observed effect upon proFMRFa. However, our results revealed that *brr2* mutants were affected in many processes and molecular functions, albeit with some prevalence for nuclear components, cytoplasm and plasma membrane components (S6 Fig). Regarding axonal guidance related genes (GO 0007411), 57 genes showed significant splicing events (S7B Fig, S7 Table), which may explain the *brr2* Tv4 axon pathfinding defects.

In addition to the splicing events detected, transcriptome analysis by RNA-Seq revealed 3,780 genes with 2-fold changed expression (up- or down-regulated) in *brr2* mutants (Fig 6D, S7 Table). From the genes related to BMP signaling pathway, only *screw* was affected. Regarding axonal guidance, 8 genes were up or downregulated in *brr2* mutants (S7B Fig, S7 Table).

Summarizing, the splicing defects observed in *brr2* mutants lead to an increase of the tkv-PD spliced form, which is less efficient with respect to the binding of the ligand. This would hamper receptor assembly, the phosphorylation of Mad and therefore the specification of Tv4 neuron. This result agrees with our previous observations, in particular the loss of pMad staining and the partial rescue of *brr2* mutants by expression of *sax*^*A*^ and *tkv*^*A*^ but not with *gbb*.

## Discussion

### Cell-specific alternative role of an ubiquitous spliceosome component

In this study, we describe a cell-specific role of *Brr2* in controlling the specification of the neuropeptidergic identity of the Tv4 neuron in the embryonic *Drosophila* Central Nervous System (CNS). This control is achieved through its role in three different steps necessary for the Tv4 neuron specification: (1) the axon pathfinding; (2) Dimm expression; and (3) activation of the BMP signalling. The role of Brr2 in the spliceosome complex has been widely studied and it is known to be essential for proper RNA processing[22, 40]. Brr2 has also been identified as a fundamental component of the alternative splicing process in *Drosophila* [17], and in addition, *brr2* expression is ubiquitous in *Drosophila* [36, 37]. These findings prompted us to anticipate a widely defective splicing pattern. However, our RNA-Seq analysis revealed that a limited number of genes were affected. Furthermore, our genetic analysis show that while *brr2* is necessary for proFMRFa and Nplp1 expression in the Ap cluster, expression of these neuropeptides in other cells in the VNC (SE2 cells for proFMRFa and dAp for Nplp1) was not affected. In addition, after a detailed analysis of pMad expression along the VNC of *brr2* mutants, we are not able to find differences with respect to the wild type pMad expression. This set of results demonstrates that the roles of ubiquitous components of the spliceosome can be highly cell-type selective.

Why Brr2 activity is restricted to promoting BMP signalling only in the Tv4 neuron of the Ap cluster, but not in other areas of the VNC, remains unclear. *tkv* and *Medea* are key components of the BMP signalling pathway and act in a number of neurons projecting out of the CNS. However, the number of pMad-positive neurons is not affected in *brr2* mutants. Protein-protein interaction has been described between Eya and Brr2 in a co-immunoprecipitation analysis in *Drosophila* [41]. The same study also described a similar interaction between Prp8 and Ap. It is tempting to speculate that these interactions could allow specific recruitment/activity of the spliceosome complex in Ap cluster cells, granting efficiency and specificity to this component of the spliceosome. The association between transcription and splicing is frequent and increases expression efficiency [42, 43]. Examples of these phenomena can be found in *Drosophila* Mef2 transcription factor family, where integration of both processes is involved in muscle fibre specification [2]. Moreover, cell-specific splicing events have been previously reported in the *Drosophila* nervous system, for instance with regards to splicing of the *Dscam* gene [44–46].

### Brr2 mutants show a specific but pleiotropic phenotype

Although the role of *brr2* in the Tv4 neuron has been delimitated with high resolution in the case of the post-transcriptional effects upon *tkv* and *Medea*, the underlying mechanisms in the axonal pathfinding defects and the lack of Dimm expression observed in Tv4 neurons remains unresolved. Regarding the pathfinding phenotype, the RNA-Seq analysis in this study revealed significant changes in 8 genes and splicing defects in 57 genes related to axon guidance. The specific role, if any, of each of these genes in the Ap cluster is unknown. Nevertheless, our study has identified clear candidate genes which, together with the well-described axonal pathfinding of Tv4, provide an excellent starting point for studying axonal guidance. Using specific mutant backgrounds and co-expression of different splicing variants identified in this study, it would be possible to shed light into the function of those genes, not only at the expression level but with regards to different isoforms, as have been previously described for the axon pathfinding gene *lola* [47, 48]. With respect to Dimm, our RNA-Seq analysis did not identify any defects in known genes which could explain the lack of its expression, which suggest that further analysis is required to identify more genes related to Nplp1 and proFMRFa neuropeptide expression.

The low efficiency of the rescue mediated via expression of active forms of *tkv* and *sax* (*apterous-Gal4>saxA*, *tkvA*) suggests that Brr2 could be regulating additional components downstream of pMad. The nuclear localization of pMad in a subset of cells where proFMRFa was not restored suggests that the splicing defects in *Medea* are not interfering with its function, at least with respect to its translocation to the nucleus. Indeed, these results match those from *in vitro* studies wherein this mRNA isoform encodes a Medea protein with apparently normal DNA-binding activity [39]. Nevertheless, its behavior *in vivo* is unknown and we cannot rule out that changes in the Medea splicing may affect the function of the Med/pMad complex in other ways, maybe related to cofactor binding as well as DNA-binding.

The specification of Tv4 neurons requires an elaborate gene cascade, which converges to activate the expression of the *FMRFa* neuropeptide gene [8, 10–14, 32]. One of the necessary components in this cascade is retrograde BMP signalling, and a number of events are necessary to activate the BMP signalling in the Tv4 neuron. Here, we demonstrate how the *brr2* gene regulates this process at three different points: Control of pathfinding of the Tv4 axon, which is necessary for BMP ligand reception and regulation of proper splicing of two crucial players of the BMP signalling pathway, *tkv* and *Medea*.

In summary, here we report post-transcriptional mechanisms that sculpt neural architecture at a cell-specific level, controlling precise aspects of cell development that together define precise and discrete cell identities. This increases our understanding of how alternative splicing is utilized during neural development. Future studies will be needed to determine if similar cell-specific splicing mechanisms is utilized in other lineages contributing to neural diversity, and emerging new technologies and bioinformatics for RNA studies will facilitate this work.

## Methods & materials

### Fly stocks

*UAS-myr-mRFP*, *FMRFa-eGFP*, *brr2*^*09c117*^*; UAS-myr-mRFP*, *FMRFa-eGFP*, *brr*^*13A036*^*; UAS-myr-mRFP*, *FMRFa-eGFP*, *Prp8*^*04p024*^ [21]. From Bloomington Drosophila Stock Center: *ap*^*md544*^ (referred to as *apGal4*, BL#3041), *elav-GAL4 2* (referred to as *elav-Gal4*, BL#8765), *UAS-mCD8GFP* (BL#8746). Other stocks used: *UAS*-*gbb*, *UAS*-*sax*^*a*^, *UAS*-*tkv*^*a*^, *btn-lacZ* [11].

Mutants were maintained over *GFP*- or *YFP*-marked balancer chromosomes. As wild type, *OregonR* or *w*^*1118*^ was used. Staging of embryos was performed according to Campos-Ortega and Hartenstein [49].

### Immunohistochemistry

Immunohistochemistry was performed as previously described [8]. Antibodies used were: guinea pig anti-Col (1:1000); guinea pig anti-Dimm (1:1000); chicken anti-proNplp1 (1:1000); rabbit anti-proFMRFa (1:1000) [13]. Mouse anti-Seven up (1:50) (Y. Hiromi, National Institute of Genetics, Mishima, Japan). Rabbit anti-pMad (1:500) (41D10, Cell Signaling Technology). Mouse mAb Eya10H6 (1:250) (from Developmental Studies Hybridoma Bank). Rabbit α-Cas (1:250) [50]. Rat α-Sqz (1:750) [51]. Rat α-Grh (1:1000) [8]. Rabbit α-Nab (1:1000) [52]. Rabbit α-Ilp7 (1:1000) (Miguel-Aliaga, Irene, London institute of Medical Sciences, Imperial College London, UK). All polyclonal sera were pre-absorbed against pools of early embryos. Secondary antibodies were conjugated with FITC, Rhodamine-RedX or Cy5 and used at 1:500 (Jackson ImmunoResearch). Embryos were dissected in PBS, fixed for 25 minutes in 4% paraformaldehyde, blocked and processed with antibodies in PBS with 0.2% Triton X-100 and 4% donkey serum. Slides were mounted with Vectashield (Vector Labs). Wild type and mutant embryos were stained and analyzed on the same slide.

### Confocal imaging and data acquisition

Zeiss LSM700 or Zeiss LSM800 confocal microscopes were used for fluorescent images; confocal stacks were merged using LSM software or Fiji software [53]. Images and graphs were compiled in Adobe Illustrator.

### RNA-sequencing and differential alternative splicing analysis

Two biological replicates of frozen collections of St15-AFT embryos control (*Orizo2*), *brr2*^*09C117*^ and *Prp8*^*04P024*^ (50 mg) were used for RNA extraction, using RNeasy Mini Kit (Qiagen, Hilden, Germany). Sample RNA yield was measured with a NanoDrop, precipitated in ethanol, and then sent to GeneWiz (GeneWiz, New Jersey, NJ) for library preparation and sequencing. Yield was checked, upon receipt of each sample, by use of NanoDrop, Qubit RNA Assay and Agilent Bioanalyzer. The samples were fragmented after RNA QC, reverse transcribed with random primers, and barcode tagged. Sequencing was performed by on the Illumina Hiseq 2500, in 1x50 bp single-read sequencing configuration (the output was stored as FASTQ-files,), which yielded 31–38 million reads/sample. The FASTQ-files were aligned against the dmel reference genome (Release 6, RefSeq GCF_000001215.4) and raw reads were normalized as reads per kilobase-length of gene per million mapped sequence reads (RPKM). Differential alternative splicing (DAS) analysis was performed as described [54–57]. Raw RNA-Seq reads were aligned to *Drosophila melanogaster* (dm3) genome using STAR (version 2.5.1b) [58] with default settings, and only uniquely mapped reads were retained to compute the number of reads for exons and exon-exon junctions in each sample using the Python package HTSeq [59], with the annotation of the UCSC Ensembl gene annotation (dm3_ensGene)[60]. Dirichlet-multinomial distribution was used to formulate the counts of the reads that were aligned to each isoform of each event [61], and the likelihood ratio test was used to test the significance of the changes in alternative splicing between *brr2* or *Prp8* mutants and controls [62]. The Benjamini-Hochberg procedure was applied to calculate the adjusted *q*-values from the *p-*values in the likelihood ratio test [63]. Seven types of differential alternative splicing events are tested, including Exon skipping (ES), Alternative 5' splice sites (A5SS), Alternative 3' splice sites (A3SS), Mutually exclusive exons (ME), Intron retention (IR), Alternative first exons (AFE) and Alternative last exons (ALE) [64]. In addition, Percent Spliced In (PSI, Ψ) was first computed to examine the percentage of the inclusion of variable exons in the exon-skipping events compared to the both isoforms [65], which is extended to evaluate splicing changes of all the seven splicing types in our DAS analysis. Particularly, the PSI was calculated as the percentage usage of the longer isoform compared to total mRNAs for ES, A5SS, A3SS, ME and IR. However, the PSI was calculated as the percentage of the usage of the proximal isoform, where the isoform with the variable exon is closer to the constitutive exon, for AFE and ALE. The differential alternative splicing events were identified under |ΔΨ| > 0.03 and *q* < 0.25.

Gene Ontology enrichment analysis was performed using GeneCodis3 for GO Biological process, GO Molecular function, and Go cellular component; lowest annotation levels, Hypergeometric statistical test, FDR P-value correction [66–68].

### Statistical analysis

Pearson’s chi-squared test, Monte Carlo, Student’s t-test, were performed using SPSS v.23 (IBM; for specific statistical test used, see text and figures). Microsoft Excel 2010 was used for data compilation and graphical representation.

## Supporting information

S1 Table Significant splicing events common to *brr2* and *Prp8* mutants (|ΔΨ| >0,03; q<0.25). Values relative to the splicing events associated with the respective gene region (FlyBase gene symbol), using the Benjamini-Hochberg procedure. ES (Exon skipping), A5SS (Alternative 5' splice sites), A3SS (Alternative 3' splice sites), ME (Mutually exclusive exons), IR (Intron retention), AFE (Alternative first exons) and ALE (Alternative last exons), Ψ (PSI, percent Spliced).
(XLSX)

S2 Table Significant splicing events in *brr2* mutants (|ΔΨ| >0,03; q<0.25). Values relative to the splicing events associated with the respective gene region (FlyBase gene symbol), using the Benjamini-Hochberg procedure. ES (Exon skipping), A5SS (Alternative 5' splice sites), A3SS (Alternative 3' splice sites), ME (Mutually exclusive exons), IR (Intron retention), AFE (Alternative first exons) and ALE (Alternative last exons), Ψ (PSI, percent Spliced).
(XLSX)

S3 Table Significant splicing events in *Prp8* mutants (|ΔΨ| >0,03; q<0.25). Values relative to the splicing events associated with the respective gene region (FlyBase gene symbol), using the Benjamini-Hochberg procedure. ES (Exon skipping), A5SS (Alternative 5' splice sites), A3SS (Alternative 3' splice sites), ME (Mutually exclusive exons), IR (Intron retention), AFE (Alternative first exons) and ALE (Alternative last exons), Ψ (PSI, percent Spliced).
(XLSX)

S4 Table Differential alternative splicing (DAS) analysis data of *brr2* mutants. Numerical data related to DAS analysis. Event_id: contains the gene cluster, chromosome, strand, position, gene ID, and exon path information. Event_type: ES (Exon skipping), A5SS (Alternative 5' splice sites), A3SS (Alternative 3' splice sites), ME (Mutually exclusive exons), IR (Intron retention), AFE (Alternative first exons) and ALE (Alternative last exons). Path1: the exonic part numbers for the first path. Path2: the exonic part numbers for the second path. logoddsB/ logoddsW: log-odds of for *brr2* mutants and control respectively, It represent the comparison between the expression level of one splicing isoform to the other. Logoddratio: the log odds-ratio between *brr2* mutants and Control. pval: p-value. qval: q-value. PSI_B: Percent Spliced In (Ψ) for *brr2*. PSI_W: Percent Spliced In (Ψ) for control. Delta_PSI: increment of Ψ when compared *brr2* to control.
(XLSX)

S5 Table Differential alternative splicing (DAS) analysis data of *Prp8* mutants. Numerical data related to DAS analysis. Event_id: contains the gene cluster, chromosome, strand, position, gene ID, and exon path information. Event_type: ES (Exon skipping), A5SS (Alternative 5' splice sites), A3SS (Alternative 3' splice sites), ME (Mutually exclusive exons), IR (Intron retention), AFE (Alternative first exons) and ALE (Alternative last exons). Path1: the exonic part numbers for the first path. Path2: the exonic part numbers for the second path. logoddsP/ logoddsW: log-odds of for *Prp8* mutants and control respectively, It represent the comparison between the expression level of one splicing isoform to the other. Logoddratio: the log odds-ratio between *Prp8* mutants and Control. pval: p-value. qval: q-value. PSI_P: Percent Spliced In (Ψ) for *Prp8*. PSI_W: Percent Spliced In (Ψ) for control. Delta_PSI: increment of Ψ when compared *Prp8* to control.
(XLSX)

S6 Table Gene expression levels 2-fold up- or down-regulated in *brr2* mutants. Values relative to the gene (FlyBase gene symbol) expression levels with 2-fold change in *brr2*, expressed as RPKM (Reads Per Kilobase per Million). The table also shows a comparison of the expression levels detected for those same genes in *Prp8* mutants. *brr2* and *Prp8* fold change is related to the control expression level. GO ID indicates gene ontology identification term/terms associated with the relative gene.
(XLSX)

S7 Table Expression levels and splicing events detected for genes with the “TGF-β receptor signaling pathway” GO identifier in *brr2* mutants. Values relative to the gene (FlyBase gene symbol) expression levels expressed as fold change, and splicing events for genes with the “TGF-β receptor signaling pathway” GO identifier in *brr2* mutants. Expression in CNS is indicated (FlyBase, BDGP). ES (Exon skipping), A5SS (Alternative 5' splice sites), A3SS (Alternative 3' splice sites), ME (Mutually exclusive exons), IR (Intron retention), AFE (Alternative first exons) and ALE (Alternative last exons).
(XLSX)

S8 Table Expression levels and splicing events detected for genes with the “axon guidance” GO identifier affected in *brr2* mutants. Values relative to the gene (FlyBase gene symbol) expression levels expressed as fold change, and splicing events for genes with the “axon guidance” GO identifier affected in *brr2* mutants. ES (Exon skipping), A5SS (Alternative 5' splice sites), A3SS (Alternative 3' splice sites), ME (Mutually exclusive exons), IR (Intron retention), AFE (Alternative first exons) and ALE (Alternative last exons).
(XLSX)

S9 Table Spreadsheet form of numerical data and summary statistics. Numerical data related to graphs and summary statistics in the Figs 1, 3, 5, S1, S4 and S5.
(XLSX)

S1 Fig Related to Fig 3. Expression of PMad in thoracic and abdominal segments of VNC in *brr2* mutants. (A-F) Expression of pMad in control (A, D) and *brr2* mutant (B,E) thoracic segments of stage AFT embryos. Eya (in thorax) and Dimm (in abdomen) immunostaining was used to identify neuropeptide expressing cells and provide a reference for delimiting the segments (dashed lines). Quantification of pMad within the segments did not show any significant difference in *brr2* mutants, when compared to control (Student’s t-test, thorax n>9 embryos per genotype; abdomen n = 3 embryos per genotype).
(EPS)

S2 Fig Ilp7 expression is not affected in *brr2* mutants. (A-D) Expression of Ilp7, Dimm, pMad and *FMRFa-GFP* reporter, in control and *brr2* (*brr2*^*09c117*^). Ilp7 expression not affected in Ilp7 neurons (dashed lines) in *brr2* mutants. Identification, using Dimm, of the previously described four Ilp7 expressing neurons (C-D dashed lines), which are BMP signaling dependent, revealed pMad expression in *brr2* mutants, indicating that the BMP pathway is active.
(EPS)

S3 Fig Related to Fig 4. Ectopic expression of the BMP ligand Gbb in *brr2* mutants. (A-B) Ap cluster neurons (white arrows), identified by *ap-Gal4>UAS-GFP*, immunostained for FMRFa and Nplp1, in control and in *brr2* mutants ectopically expressing *gbb* (*ap>gbb;brr2*). Transgenic expression of *gbb* does not result in rescue of FMRFa expression.
(EPS)

S4 Fig Related to Fig 5. Characterization of FMRFa rescue. (A-C) Expression of *FMRFa>GFP* (FMRFa), *ap>RFP* and pMad, in *ap-Gal4;brr2* mutants (*brr2*^*09c117*^) transgenically expressing *sax*^*A*^ and *tkv*^*A*^, at AFT. pMad expression is rescued in the Ap cluster by ectopic expression of active forms of Tkv and Sax, although FMRFa expression is not restored in every segment (B). (A) and (C) show magnification of squares “1” and “2” depicted in (B). More than one cell in the Ap cluster shows expression of pMad (variable expression level). (D-E) Expression of *FMRFa>GFP* (FMRFa), *ap>RFP* reporter and Dimm in *ap-Gal4;brr2* mutants (*brr2*^*09c117*^) transgenically expressing *sax*^*A*^ and *tkv*^*A*^, at AFT. FMRFa rescue does not correlate with Dimm expression (arrows). (F) Quantification of Dimm expressing cells in control, *brr2* mutants and *ap-Gal4;brr2* mutants (*brr2*^*09c117*^) expressing *sax*^*A*^ and *tkv*^*A*^ (*ap-Gal4 sax[A]* and *tkv[A]; brr2*; Pearson’s chi-squared test, n>34 Ap clusters per genotype, * = p<0.05; *** = p<0.001). (G-H) Expression of *FMRFa>GFP* (FMRFa), *ap>RFP* and pMad, combined with DAPI staining, in *ap-Gal4;brr2* mutant (*brr2*^*09c117*^) expressing *sax*^*A*^ and *tkv*^*A*^, T2 segments, right and left of the same embryo. pMad nuclear localization appears to be independent of FMRFa rescue. (I) pMad expressing cluster quantification (referred as Ap cluster with at least one strong pMad expressing cell) in control, *brr2* mutants and *ap-Gal4;brr2* mutants (*brr2*^*09c117*^) expressing *sax*^*A*^ and *tkv*^*A*^ (*ap-Gal4 sax*^*A*^ and *tkv*^*A*^*;brr2*; Pearson’s chi-squared test, n>34 Ap clusters per genotye; *** = p<0.001).
(EPS)

S5 Fig Related to Fig 6. *Prp8* phenocopies *brr2* mutants. (A-F) Confocal, 2–5 layer projection images of the Ap cluster showing expression pattern of the late factors in the specification cascade by immunostaining of Eyes absent (Eya), Dimmed (Dimm) and phosphorylated form of Mothers against dpp (pMad) in *Prp8* mutants (*Prp8*^*04p024*^; E-F) compared to the pattern previously observed in control (A-B) and *brr2* mutant *(brr2*^*09c117*^*/ brr*^*13A036*^; C-D) embryos, at stage AFT. (G) Quantitative analysis of the SE2 FMRFa cells size in control, *brr2* mutant *(brr2*^*09c117*^*/ brr*^*13A036*^), *ap-Gal4;brr2* mutants (*brr2*^*09c117*^) expressing *sax*^*A*^ and *tkv*^*A*^ (*ap> sax*^*A*^ and *tkv*^*A*^*;brr2) and elav-Gal4;brr2* mutants (*brr2*^*09c117*^) expressing *sax*^*A*^ and *tkv*^*A*^ (*elav> sax*^*A*^ and *tkv*^*A*^*;brr2*).
(EPS)

S6 Fig Related to Fig 6. Top Gene Ontology terms affected in *brr2* mutants. (A) Gene Ontology (GO) enrichment analysis of genes showing splicing events in *brr2* mutants (GeneCodis). (B) Gene Ontology (GO) enrichment analysis of genes showing 2-fold transcription changes in *brr2* mutants (GeneCodis).
(EPS)

S7 Fig Related to Fig 6. Top Gene Ontology terms affected in *brr2* mutants (b). (A) Gene Ontology (GO) enrichment analysis of genes showing both splicing events and 2-fold transcription changes in *brr2* mutants (GeneCodis). (B) Venn diagram showing axon guidance affected genes in *brr2* mutants.
(EPS)

S1 Video Related to Fig 4. Dorsal neurohemal organ inervation by apterous cluster neurons in control. 3D reconstruction video from confocal images showing the axonal pathfinding of Ap cluster neurons (ap>GFP, green) innervating the dorsal neurohemal organ (DHN, *buttonless-lacZ*, blue) in the thoracic segments T2-T3 of control flies at stage AFT.
(MP4)

S2 Video Related to Fig 4. Dorsal neurohemal organ innervation failure by apterous cluster neurons in *brr2* mutants. 3D reconstruction video from confocal images showing the axonal pathfinding of Ap cluster neurons (ap>GFP, green) and the absence of innervation to the dorsal neurohemal organ (DHN, *buttonless-lacZ*, blue) in the thoracic segments T2-T3 of *brr2* mutant flies at stage AFT.
(MP4)

S3 Video Related to Fig 5. Tv4 neuron axonal pathfinding in control. 3D reconstruction video from confocal images showing the axonal pathfinding of the Ap cluster (ap> RFP, red) and the Tv4 neurons (FMRFa>GFP, green) in the thoracic segment T3 of control flies at stage AFT. The reconstruction shows Tv4 projection to the midline and then dorsal, establishing a ramified pattern (DHN inervation).
(MP4)

S4 Video Related to Fig 5. Tv4 neuron axonal pathfinding in *brr2* mutants with rescued FMRFa expression. 3D reconstruction video from confocal images showing the axonal pathfinding of the Ap cluster (ap> RFP, red) in *brr2* mutants rescued by transgenic expression of activated BMP receptors (*sax*^*A*^ and *tkv*^*A*^). Expression of *FMRFa-eGFP* reporter make possible to identify the Tv4 neuron. The segment shown (T2) present partial rescue of FMRFa expression on one cluster (FMRFa>GFP, green, at the starting frame, cell located on the right side) but not in the other (left side). The reconstruction shows Tv4 cell projecting their axon normally towards the midline, but fails to project dorsally into the DNH. Instead, the axon follows the same trajectory as the rest of the Ap cluster neurons (*ap>RFP*), projecting ipsilaterally and anteriorly.
(MP4)
